# Departments Which Do Not Always Pay

**Published:** 1912-05-25

**Authors:** 


### INSTITUTIONAL HOUSEKEEPING.

### Departments Which Do Not Always Pay.

### III.—THE PREPARATION OF BEEF-TEA.

In large hospitals, infirmaries, and mental hospitals beef-tea is made on a scale which raises it to the dignity of a separate department of kitchen economy. It is rather remarkable that this form of administering nourishment still continues popular with doctors, nurses, and patients, though it has long been discredited in the eyes of the economist and of the physiologist. The very poor value of ordinary beef-tea in heat-producing or body-building substances has been exposed over and over again. It would seem that it is not quite such an indispensable feature as is generally supposed in hospital expenditure, for managers practised in the art of keeping down expenses have in many instances successfully demonstrated that institutions for the sick can get on very well without this costly item in the diets. And yet from force of habit it continues to play an important part in the dietary system of invalids. It certainly has a stimulating and cheering effect on patients who can take very little else, and for this cause it is likely to remain a leading feature in the list of "extras."

To make this department pay it is necessary to procure the meat from which it is made at the lowest possible cost, and to extract from it every particle of its valuable contents. In both these directions serious blunders are made by stewards, matrons, and cooks. The present unprecedentedly high price of beef has a very important bearing on the cost of this department. A few years ago it was possible to procure sound foreign shin of beef for the preparation of beef-tea at about 2d. or 2½d. a pound. The tea brewed from this description of meat was found when analysed to contain fully as much nutritive matter and extractives as that brewed from the best English shin. But in the present condition of the meat market the same quality of foreign meat costs quite twice as much. With this rise in price the question presents itself more forcibly than before: Does the process adopted for brewing the tea succeed in extracting the entire value from the meat? This is a question not to be answered offhand in the affirmative by any manager or housekeeper. There may be a strong impression that the residue is worthless, while yet a large proportion of the best properties of the meat lie latent in the material which is thrown away. That this is the case in many old-fashioned hospital kitchens with their rough-and-ready way of "stewing down" the beef can hardly be doubted. Let managers who wish to assure themselves that their method of making beef-tea pays, go to the trouble of having the residue analysed after the infusion has been made.

In some of the very large institutions special apparatus is in use for this business of making beef-tea. The apparatus in use at Claybury Mental Hospital was described in a recent number of The Hospital. Even when scientific methods of extracting the "goodness" of the beef are in use it is necessary to inquire whether the trouble of making beef-tea is compensated by any saving as compared with the concentrated article. Where proper appliances are at the disposition of the cook, and where meat can be contracted for at low rates, the tea may be brewed at a price which makes it worth while to undertake its manufacture. In smaller institutions there is reason to believe that the use of some concentrated form of beef-tea of proved excellence would be a source of considerable saving, not merely in trouble but in money also. Those who doubt this are recommended to measure pint for pint the cost and quality of home-made as compared with concentrated beef-tea. It will be found that strength for strength the concentrated article has usually the advantage, and there is therefore no particular reason why the labour and risk of attempting to prepare beef-tea in the kitchen should be attempted.

There is another consideration which ought to weigh with the economist. To substitute the concentrated article for the home-made is to remove beef-tea definitely from its position as an ordinary article of diet to that of a prescription. It is frequently used as a kind of soporific for supper when its place might far more economically be taken by broth made from a carefully strained hospital stock-pot. To use the concentrated article only whenever beef-tea is prescribed is to diminish very considerably the quantity required in the establishment.

Where insufficient precautions are taken, either to limit the quantity of beef-tea used or to buy the meat at a low price, or to extract from it all its nourishing properties in the process of making the tea, it is only too clear that hundreds of pounds' worth of good material can be wasted yearly over this simple hospital drink.

### Housekeeping Queries.

#### Preserving Meat in Warm Weather.

Housekeeper.—*Our cottage hospital is very isolated, and we can only obtain fresh meat once a week. In warm weather it is very difficult to keep it, and I should be so glad of directions for pickling beef so that I can feel independent of weather and butcher. The latter's salt beef is not nice.*

A useful brine for pickling beef is made as follows: Put into a large saucepan 1 lb. salt, 6 oz. of brown sugar, 1 oz. of saltpetre, and 4 quarts of water. Boil these for ten minutes, keeping the liquid well skimmed. Strain it off into a large basin or any earthenware vessel, and when perfectly cold lay in the meat. Before the beef is laid in cut away any veins or kernels in it. Be very careful that the meat is kept under the brine, if necessary put a weight on it. Leave in pickle for about ten days for a thick piece. The saltpetre reddens the meat; if too much is used or the meat is kept in too long it will be hard. This brine can be used again several times if strained, reboiled, well skimmed, and about 4 oz. more salt added. Pig's head or pork can also be pickled in this brine.

